# Presence of secondary bladder cancer following radical nephroureterectomy for upper tract urothelial carcinoma: characteristics, risk factors, and predictive value

**DOI:** 10.1186/s12894-022-01158-6

**Published:** 2022-12-24

**Authors:** Shicong Lai, Pengjie Wu, Shengjie Liu, Samuel Seery, Jianyong Liu, Lei He, Ming Liu, Yaoguang Zhang, Jian-ye Wang, Tao Xu

**Affiliations:** 1 Department of Urology, Peking University People’s Hospital, No. 11 Xizhimen South Street, Beijing, 100044 China; 2 Graduate School of Peking Union Medical College, Chinese Academy of Medical Sciences, Beijing, 100730 China; 3 Department of Urology, Beijing Hospital, National Center of Gerontology, Institute of Geriatric Medicine, Chinese Academy of Medical Sciences, No. 1 DaHua Road, Dong Dan, Beijing, 100730 China; 4 School of Humanities and Social Sciences, Chinese Academy of Medical Sciences and Peking Union Medical College, Beijing, 100730 China; 5 Department of Health Research, Faculty of Health and Medicine, Lancaster University, Lancaster, LA1 4YW UK; 6 Department of Pathology, Beijing Hospital, National Center of Gerontology, Institute of Geriatric Medicine, Chinese Academy of Medical Sciences, Beijing, 100730 China

**Keywords:** Cancer-specific survival, Radical nephroureterectomy, Secondary bladder cancer characteristics, Upper urinary tract urothelial carcinoma

## Abstract

**Background:**

To assess the characteristics, predictive risk factors, and prognostic effect of secondary bladder cancer (SBCa) following radical nephroureterectomy (RNU) in upper tract urothelial carcinoma (UTUC).

**Methods:**

Using the Surveillance, Epidemiology, and End Results database, the authors analyzed clinicopathologic characteristics and survival data from 472 UTUC patients with SBCa after RNU, between 2004 and 2017. Cox’s proportional hazard regression model was implemented to identify independent predictors associated with post-recurrence outcomes. The threshold for statistical significance was *p* < 0.05.

**Results:**

In total, 200 Ta-3N0M0 localized UTUC patients with complete data were finally included. With a median follow-up of 71.0 months (interquartile ranges [IQR] 36.0 -103.8 months), 52.5% (n = 105) had died, with 30.5% (n = 61) dying of UTUC. The median time interval from UTUC to SBCa was 13.5 months (IQR 6.0–40.8 months). According to multivariable Cox regression analysis, patients with SBCa located at multiple sites, advanced SBCa stage, higher SBCa grade, elderly age and a shorter recurrence time, encountered worse cancer-specific survival (CSS), all *p* < 0.05.

**Conclusion:**

For primary UTUC patients with SBCa after radical surgery, advanced age, multiple SBCa sites, shorter recurrence time, higher SBCa stage, and grade proved to be significant independent prognostic factors of CSS. We ought to pay more attention to SBCa prevention as well as to earlier signs which may increase the likelihood of early detection. Having the ability to manage what may be seen as the superficial SBCa signs may enable us to improve survival but further research is required.

## Background

Upper urinary tract urothelial carcinoma (UTUC) is a lethal malignancy which has characteristic synchronous or metachronous multifocal recurrences throughout the genitourinary tract [1–3]. Radical nephroureterectomy (RNU) with bladder cuff excision (BCE) remains the gold standard surgical intervention for non-metastatic UTUC; however, there are still 20–50% who develop secondary bladder cancer (SBCa) following radical surgery throughout their lifetime [4]. Some have even reported that more than 60% of those undergoing RNU will develop SBCa postoperatively within two years.

Numerous studies have been designed to identify the relevant SBCa predictors after RNU. However, little detailed data around the clinicopathologic characteristics, predictive risk factors, and prognostic effect of SBCa in UTUC exists. Yamashita et al. and Elalouf et al. found that patients with SBCa after RNU had worse cancer-specific survival (CSS) and indeed overall survival [5, 6]. Unfortunately, this evidence comes from seemingly disparate studies, involving relatively few participants which inhibits our ability to provide strong recommendations. Hou et al. once developed a prognostic nomogram using a clinical data set garnered from the surveillance, epidemiology and end results (SEER) database between 2010 and 2015 [7]. However, despite the analytical sophistication involved in their study, it suffered substantial recruitment bias and incomplete individual patient data which decreases the value and reduces reliability. Additionally, a large proportion of UTUC patients included in their final sample did not undergo bladder cuff excision (BCE) which may actually predispose patients to local recurrences [8, 9].

Complete information around histopathological characteristics is necessary if we are to develop a reliable evidence-base to guide best clinical practice. However as yet, few have managed to design, conduct, report and disseminate investigations which garner real insight for clinical practice. As such, it remains necessary to conduct a rigorous assessment of factors associated with post-recurrence outcomes in primary UTUC patients with SBCa following radical surgery.

## Methods

### Data source and study population

After approval from the National Cancer Institute (NCI), patients diagnosed with UTUC (renal pelvis or ureteral) between 2004 and 2017 were identified from nine SEER cancer registries. The site codes C65.9 and C66.9 as well as histological codes taken from the 3rd edition of International Classification of Diseases for Oncology (i.e., 8120, 8122, 8130, and 8131) were used to identify patients. All selected patients underwent RNU with BCE and subsequently had received SBCa diagnosis.

Patients were deemed ineligible if they met *any* of the following criteria: (1) Patients with synchronous or previous BCa when diagnosed with UTUC; (2) Patients with bilateral UTUC; (3) Patients with distant metastatic UTUC or only with lymph node metastasis; (4) patients who had not undergone radical surgery or had received RNU without BCE; (5) Patients whom did not develop SBCa post-surgery; and (6) Patients with insufficient clinicopathological information or incomplete survival data (e.g., ethnicity, gender, tumor location, tumor stage, grade, architecture, laterality, cause of death, interval between UTUC and SBCa, length of survival, and unknown length of follow-up).

Since the SEER database is publicly accessible and patient information was anonymous, this study does not require informed consent. However, current study conformed to the 1964 Helsinki Declaration (and the more recent amendments) and was performed in accordance with the ethical standards of the institutional and national research committee.

### Definition of variables and follow‑up

All prerequisite information was abstracted from SEER database. Basic demographics including age, gender, race, the time interval from UTUC to SBCa, and tumor characteristics (i.e., tumor location, stage, grade and architecture) of both UTUC and SBCa were carefully retrieved. As tumors histopathological grades were classified as four types (i.e., well-differentiated, moderately-differentiated, poorly-differentiated, and undifferentiated) in the SEER database, we defined the well and moderately differentiated as low grade, and defined the poorly and undifferentiated type as high grade.

**Fig. 1:**
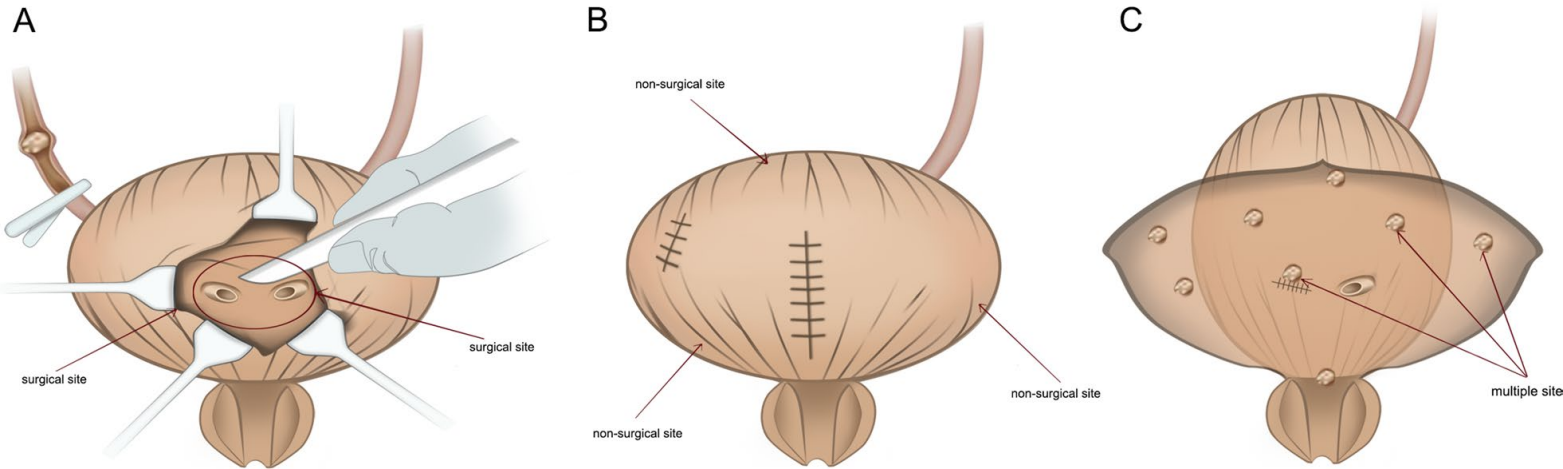
**A** An overview of the surgical site of the bladder (i.e., the bladder trigone or posterior wall) and/or open excision filed (anterior wall); **B** An overview of non-surgical site of the bladder (i.e., bladder dome, neck or later wall); **C** An overview of the multiple bladder recurrence site

The locations of SBCa were also divided into three sites: surgical site, which refers to SBCa located in BCE field (i.e., the bladder trigone or posterior wall) and/or open excision filed (anterior wall) (Fig. 1A); non-surgical site, which refers to SBCa located in the bladder dome, neck or later wall (Fig. 1B); whilst multiple sites was defined as the synchronous presence of two or more pathologically confirmed SBCa (Fig. 1C).

Recurrence time was defined as the time interval between UTUC and SBCa. CSS was defined as the time from diagnosis until death due to UTUC, which was defined according to SEER-specific cause of death coding (i.e., code 29020). Data from patients who died of other causes and those who survived to the final follow-up point were classified as censored for analytical purposes.

### Statistical analysis

Categorical variables are presented as frequencies and proportions, while continuous variables are expressed as medians with interquartile ranges (IQR). Survival curves were generated using the Kaplan–Meier method and multivariate survival analysis was assessed using Cox’s proportional hazard regression model, which is a forward step likelihood ratio based model used to identify independent risk factors associated with mortality. All analyses were conducted using the Statistical Package of Social Sciences for Windows, version 20 (SPSS, Chicago, IL). *p* values lower than 0.05 were considered statistically significant.

## Results

### Baseline characteristics of the study population

Based on predefined eligibility criteria, no pT4 patients were included in this study. A total of 200 Ta-3N0M0 localized UTUC patients met the predetermined eligibility criteria were eventually included in the final analysis. Detailed demographics and clinicopathological characteristics are presented in Table 1. In this instance, the majority of this sample were caucasian (91.0%). Overall, 132 (66.0%) patients had renal pelvic tumors and 68 (34.0%) with ureteral tumors. High tumor grade was identified in 75.5% (n = 151) patients. The pathologic stage distribution of UTUC in this cohort was 45% (n = 90) pTa-1, with 21.5% (n = 43) pT2, and 33.5% (n = 67) pT3.

Of the 200 patients in the entire cohort, the median recurrence time was 13.5 months (IQR 6.0–40.8 months). SBCa were located as follows, with 21% (n = 42) and 26.5% (n = 53) positioned at site I and II respectively. The remaining 52.5% (n = 105) who had multifocal tumors residing in site III. High tumor grade was found in 59% (n = 118) patients. Pathologic stage distribution highlighted that Non-invasive bladder cancer (NIVBC) (pTa-1) and IVBC (pT2-4) was found to be 90% (n = 180) and 10% (n = 20), respectively.

### Survival outcomes and predictors

**Fig. 2:**
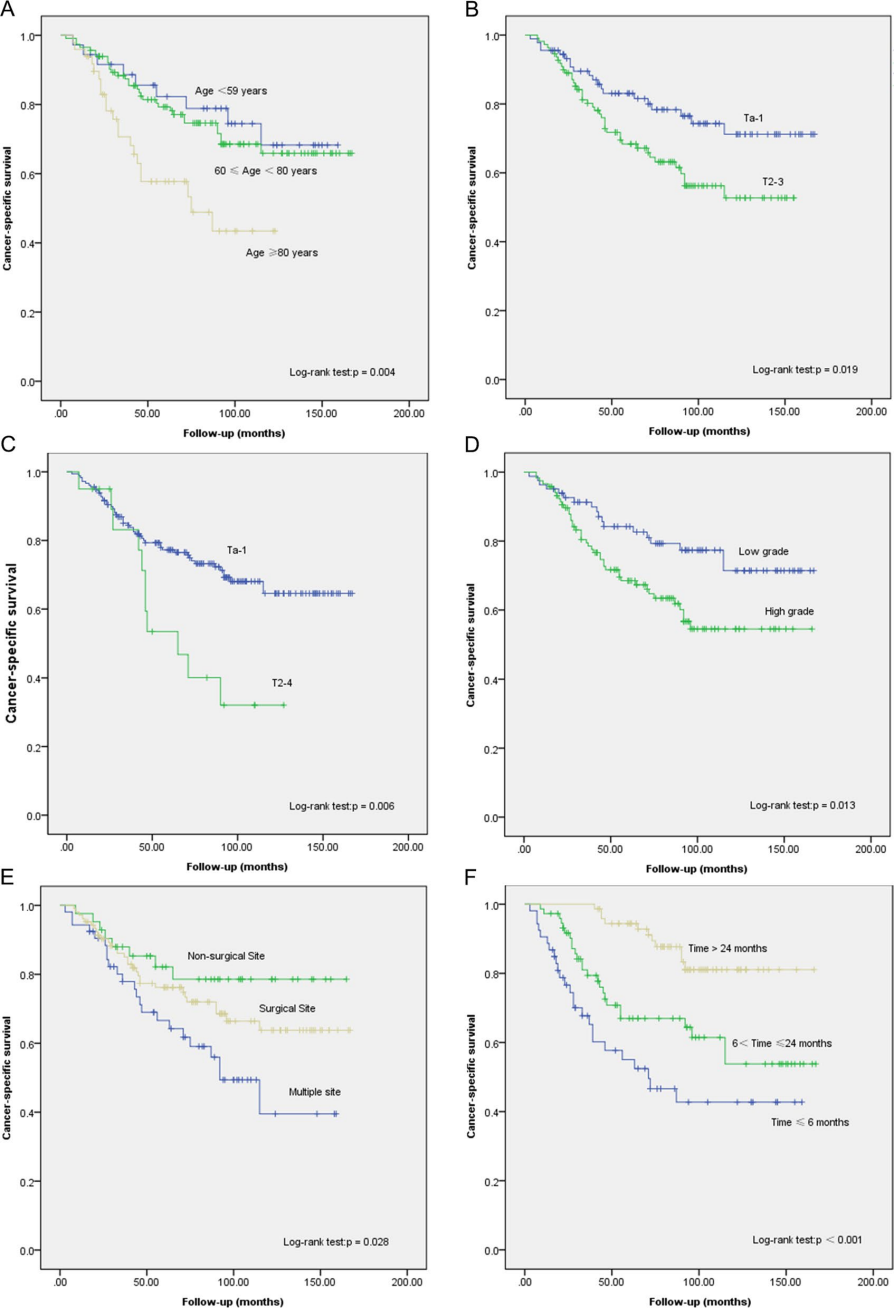
Cancer-specific survival of patients with SBCa following radical nephroureterectomy stratified by **A** Age, **B** UTUC stage, **C** SBCa stage, **D** SBCa grade, **E** SBCa location, and **F** Recurrence time. *UTUC* Upper urinary tract urothelial carcinoma, *SBCa* Secondary bladder cancer

At the median follow-up of 71.0 months (IQR 36.0–103.8 months), 52.5% (n = 105) had died, of whom 30.5% (n = 61) had died of UTUC. As can be seen in Table 2; Fig. 2, both Kaplan–Meier and univariable Cox regression analyses suggested that patient age, UTUC stage, SBCa stage, SBCa grade, SBCa location, and recurrence time are significant, independent predictors of CSS. However, under multivariable Cox regression analysis, UTUC tumor stage was not an independent predictive factor of CSS.

**Table 1:** Characteristics of patients with SBCa following RNU for non-metastatic UTUC

Variables	Number of cases
Age in years, n (%)	
< 60	36 (18.0)
60–79	115 (57.5)
≥ 80	49 (24.5)
Gender, n (%)	
Male	113 (56.5)
Female	87 (43.5)
Race, n (%)	
White	182 (91.0)
Black	6 (3.0)
Other	12 (6.0)
UTUC laterality, n (%)	
Left	103 (51.5)
Right	97 (47.5)
UTUC location, n (%)	
Pelvis	132 (66.0)
Ureter	68 (34.0)
UTUC stage, n (%)	
pTa-1	90 (45.0)
pT2-3	110 (55.0)
Histopathological UTUC, n (%)	
Low (well and moderately differentiated)	49 (24.5)
High (poorly differentiated and undifferentiated)	151 (75.5)
SBCa location, n (%)	
Non-surgical Site (bladder dome, neck or later wall)	42 (21.0)
Surgical site (bladder trigone, anterior or posterior wall)	53 (26.5)
Multiple site (multifocal tumors)	105 (52.5)
SBCa stage, n (%)	
pTa-1	180 (90.0)
pT2-4	20 (10.0)
Histopathological SBCa grade, n (%)	
Low (well and moderately differentiated)	82 (41.0)
High (poorly differentiated and undifferentiated)	118 (59.0)
Recurrence time in months, n (%)	
Time ≤ 6	53 (26.5)
6 < Time ≤ 24	74 (37.0)
Time > 24	73 (36.5)
Follow–up in months, median (IQR)	71 (36.0, 103.8)

*RNU* Radical nephroureterectomy, *SBCa* Secondary bladder cancer, *pT* Pathological tumour, *UTUC* Upper tract urothelial carcinoma

**Table 2:** Univariate and multivariate Cox regression analyses predicting CSS for patients with SBCa following RNU for non-metastatic UTUC

Variables	Univariate			Multivariate		
	HR	95% CI	*p*	HR	95% CI	*p*
Age in years			0.006			0.021
< 59	1	Reference		1	Reference	
60–79	1.188	0.565–2.497	0.650	1.298	0.607–2.774	0.501
≥ 80	2.721	1.236–5.990	0.013	2.643	1.166–5.989	0.020
Gender						
Male	1	Reference				
Female	1.213	0.733–2.008	0.453			
Race			0.401			
White	1	Reference				
Black	0.479	0.066–3.461	0.465			
Other	0.436	0.106–1.784	0.248			
UTUC laterality						
Left	1	Reference				
Right	1.029	0.622–1.701	0.911			
UTUC location						
Pelvis	1	Reference				
Ureter	1.362	0.820–2.263	0.233			
UTUC stage						
pTa-1	1	Reference				
pT2-3	1.872	1.095–3.200	0.022			
Histopathological UTUC grade						
Low (well and moderately differentiated)	1	Reference				
High (poorly differentiated and undifferentiated)	1.764	0.918–3.391	0.089			
SBCa location			0.033			0.048
Non-surgical Site	1	Reference		1	Reference	
Surgical site	0.376	0.168–0.840	0.017	0.539	0.238–1.221	0.539
Multiple site (multifocal tumors)	0.590	0.343–1.018	0.058	0.505	0.287–0.889	0.018
SBCa stage						
pTa-1	1	Reference		1	Reference	
pT2-4	2.441	1.267–4.702	0.008	2.900	1.441–5.839	0.003
Histopathological grade of SBCa						
Low (well and moderately differentiated)	1	Reference		1	Reference	
High (poorly differentiated and undifferentiated)	1.984	1.141–3.449	0.015	1.991	1.130–3.508	0.017
Recurrence time, months			< 0.001			< 0.001
Time ≤ 6	1	Reference		1	Reference	
6 < Time ≤ 24	0.567	0.326–0.989	0.045	0.548	0.307–0.980	0.042
Time > 24	0.184	0.090–0.375	< 0.001	0.129	0.060–0.278	< 0.001

*CSS* Cancer-specific survival, *pT* Pathological tumour, *HR* Hazard ratio, *CI* Confidence interval, *SBCa* Secondary bladder cancer, *RNU* Radical nephroureterectomy, *UTUC* Upper urinary tract urothelial carcinoma

An advanced SBCa stage (hazard ratio [HR] 2.900; 95% confidence interval [CI] 1.441–5.839; *p* = 0.003) and higher SBCa grade (HR:1.991; 95% CI 1.130–3.508; *p* = 0.017) were significantly associated with worse CSS. Additionally, patients with multiple SBCa sites also had a shorter survival (*p* = 0.048). Regarding the impact of recurrence time on oncological outcomes, we defined time ≤ 6 months as the reference juncture, and findings suggest that the HR of CSS significantly decreases between 6.0 and 24.0 months (HR: 0.548; 95% CI 0.307–0.980; *p* = 0.042) and in the > 24 months group (HR: 0.129; 95% CI 0.060–0.278; *p* < 0.001). Using ≤ 59 years as the reference, we observed a decreased HR for CSS at ≥ 80 years (HR:2.643, 95% CI 1.166–5.989; *p* = 0.020) patient group. This suggests that the older age range is also an independent predictive factor for poorer CSS. Please see Table 2 for further details.

## Discussion

Despite the fact that UTUC patients with SBCa following RNU have poorer CSS, detailed descriptions of the characteristics of SBCa and their influence on post-recurrence outcomes is unknown [5, 6]. Previous research in this field has to some extent lacked rigour and therefore recommendations remain vague. In order to address this issue and add to the small but growing evidence base around prognostics for patient counselling and follow-up scheduling, we screened and selected a target population by controlling for other known prognostic factors. Using the anonymized, publicly available SEER database, we identified and analyzed data from 200 suitable candidates after considering eligibility criteria. While this sample was still relatively small we managed to garner several insights which could be useful in guiding clinical practice and indeed drive research forward in this field.

Past research has consistently suggested that, tumor stage and lymph node status are the most powerful predictors of survival in UTUC patients [10]. However, this sample univariable analysis suggests that both tumor stage for the original UTUC as well as for SBCa are important indicators in terms of survival. Although interestingly, the association between UTUC stage and survival is lost and SBCa stage becomes the more decisive factor for CSS under multivariable analysis. This ascertain can be clearly observed through statistical collinearity between UTUC and SBCa staging variables. Although, there is one addendum because this observation may be attributable to the fact that all lymph node positive patients were excluded from this sample. Additionally, there were no pT4 patients and the proportion of pT3 stage patients in this cohort was relatively low. Indeed, only one third of this sample (33.5%) were initially assessed as having pT3 tumors. Furthermore, previous studies have found that the patients with pT0-Ta-T1 carcinomas have similar CSS probabilities compared to patients with pT2 carcinomas [11, 12], although this is an area which requires further research. The other reason for this may be that our study focused on a special UTUC population. This population had developed SBCa following RNU and in all fairness some believe that SBCa should be more appropriately categorized as UTUC progression. Current results appear to show that the aggressiveness of SBCa, which is not indicated through UTUC staging, should be a priority during patient-practitioner consultations. Rationally speaking, there are likely to be distinctions between different sub-groups, related to biological behavior as well as specific risk factors associated with this type of carcinoma. However, future well-designed research is needed to develop the evidence base.

The other standard pathological feature of SBCa aggressiveness which emerged through this study was tumor grade. According to some literature, low grade SBCa poses very low risk to patients in terms of disease progression and CSS [13]. Although, this more general applies to early diagnosis which is not always possible. In high grade tumors, Pietzak et al. found that even Ta and T1 tumors share chromosomal mutations with carcinoma which invade the bladder muscle [14]. This suggests there is likely to be a fundamental difference when it comes to prognosis for BCa patients with different tumor grades. In this study, the advanced grade group also proved to be significantly associated with worse CSS. Therefore, while early diagnosis remains of the utmost importance, we must be particularly vigilant when determining tumor stage and we must discuss with patients adjuvant therapies such as immediate instillation of intravesical chemotherapy following surgery as a necessary preventative measure.

Several investigators have found a tendency for SBCa to be located on or near the resection or BCE site, where the bladder urothelium was injured during cystotomy [5, 15]. These observations are supported by findings from an in vitro experiment where researchers have found that tumor cells floating in the bladder are more likely to adhere to an injured urothelial surface [2]. However, the research, both basic and clinical, have not always yielded consistent conclusions. For example, Belhadj et al. conducted a large retrospective study which involved 24 centers and 163 patients, in order to assess the distribution of SBCa locations following RNU [1]. However, they were unable to identify a trend or even detect a preferred site of recurrence, and our findings appear consistent with this observation. There does not appear to be a significant difference in terms of recurrence rate between surgical and non-surgical locations.

As previously mentioned, detailed information around multiple SBCa for this study cohort was not available via the SEER database. Consequently, these patients were categorized as the multiple site group. When exploring the effects of these categories on oncological outcomes, multiple recurrences was determined to be a significant risk factor for worse CSS. Although, this was to be expected because multiplying tumor cells and tumors with multifocal features are of course associated with higher proliferation, migration and the ability to invade vulnerable adjoining locations.

We also found a shorter recurrence time to be associated with worse survival outcome which appears to validate the pathophysiologic mechanism hypothesis previously described. Further support for this can be found in the existing evidence base. For example, Mitra et al. conducted a study which involved 2029 patients in an attempt to identify prognostic factors for survival after urothelial recurrence following radical cystectomy for BCa [16]. The researchers found that patients experiencing early urothelial recurrence often face worse prognosis. This further highlights the aggressive nature of urothelial recurrence of urothelial carcinomas and necessitates SBCa prevention after RNU. Of course, as always, early detection and even developing effective management strategies for seemingly superficial BCa might significantly decrease tumor progression [17]. Therefore, routine cystoscopy after nephroureterectomy is important for patients in terms of postoperative management. While beyond the scope of this article to discuss this in great detail, it is important to note that this involves raising public awareness and perhaps training general practitioners to inquire when performing standardized health checks which ought to take place regularly.

Interestingly, our findings are line with findings from a 13-center study involving 1453 patients treated with RNU, those aged ≥ 60 years and also ≥ 80 years are significantly associated with decreased all-cause survival and CSS [18]. Likewise, Chromecki et al. found that patients with an advanced age at the time of RNU are significantly associated with inferior survival after radical surgery [19]. Again predictably, this effect can be attributed to the aging process where co- and indeed multiple morbidities develop, decreasing a patient’s natural defense mechanisms. There are a great many unknowns in this situation, all of which influence choice of interventions and care cycles [18, 20]. Additionally, biologic changes of the host–tumor relationship in different age groups may also influence survival [21]. Consequently, age ought to be considered when developing adjuvant therapies and patient management schedules.

While this study provided some interesting findings there are several limitations ought to be mentioned. Firstly, this retrospective study summarized data from 9 centres which provide information to the SEER database. This may mean there was selection bias and information biases which may have skewed our findings. Second, as with most previous published studies based upon the SEER database, some limitations could be attributed to the database itself. Necessary information regarding physical condition, disease focality, status and the extent of lymph node dissection, bladder instillation and other adjuvant treatments after RNU, may have influenced patients’ prognoses. Unfortunately, these factors are currently unavailable through the SEER database which constrained our analysis and has therefore inhibited our ability to provide strong guidelines. It is hoped that as the SEER database develops into a more sophisticated data source, the proprietors will incorporate more necessary variables.

## Conclusion

For primary UTUC patients with SBCa after radical surgery, advanced age, multiple SBCa sites, shorter recurrence times, higher SBCa stage and grade were significant independent prognostic factors of poorer CSS. The clinical implications are that we ought to pay more to SBCa prevention as well as to earlier signs which may increase the likelihood of early detection. Having the ability to manage what may be seen as the superficial signs of SBCa may enable us to improve survival but further research is required.

## Data Availability

The data of the current study are extracted from the SEER database.

## Abbreviations

BCE: Bladder cuff excision
BCa: Bladder cancer
RNU: Radical nephroureterectomy
SBCa: Secondary bladder cancer
UTUC: Upper tract urothelial carcinoma
SEER: Surveillance, epidemiology, and end results database
IQR: Interquartile ranges
CSS: Cancer-specific survival
NCI: National Cancer Institute
HR: Hazard ratio
CI: Confidence interval
NIVBC: Non-invasive bladder cancer
